# Reducing Weekend Hospital Discharge Delays Without Seven-Day Coverage by Leveraging Thursday and Friday Planning

**DOI:** 10.7759/cureus.87526

**Published:** 2025-07-08

**Authors:** George Bechir

**Affiliations:** 1 Hospital Medicine, Franciscan Health, Munster, USA

**Keywords:** case management, discharge planning, emergency department admissions, hospital throughput, length of stay, multidisciplinary rounds, pre-weekend discharge, seven-day coverage, weekend bottlenecks, weekend effect

## Abstract

Weekend discharge delays remain a significant contributor to prolonged hospital length of stay, often resulting from reduced availability of consultants, imaging, case management, and therapy services on Saturdays and Sundays. Expanding hospital operations to full seven-day coverage is often proposed. However, such solutions are frequently cost-prohibitive and have shown inconsistent benefits in general inpatient settings. This review proposes a more practical and scalable alternative that focuses on leveraging Thursday and Friday as high-impact planning days to mitigate weekend-related discharge bottlenecks. Through a narrative review of 15 studies published between 2010 and 2024, we identified key operational strategies, including early multidisciplinary rounds, Friday discharge huddles, and coordination guided by standardized discharge checklists, that enable teams to proactively clear discharge barriers before the weekend. These interventions improve weekend discharge volume, reduce unnecessary hospital days, and enhance patient flow without requiring full weekend staffing models. In addition, embedding case management within the emergency department can reduce avoidable weekend admissions by directing low-acuity patients toward outpatient services and follow-up. Together, these targeted weekday efforts form a cost-effective, immediately actionable framework that hospitals can adopt to reduce weekend delays, improve efficiency, and lower overall length of stay without compromising safety or quality of care.

## Introduction and background

Weekend delays remain a persistent challenge in hospital operations, frequently extending patient length of stay. Reduced availability of consultants, imaging services, interventional procedures, case management, and therapy services on weekends creates discharge bottlenecks that keep medically ready patients in house longer [1]. Case management, defined as the coordination of care and discharge planning by dedicated staff, and "social admits", referring to patients hospitalized primarily due to lack of safe outpatient support, are common contributors to these delays. Expanding seven-day service coverage can help, but such solutions are not always cost-effective or sustainable across all departments [2].

The purpose of this review is to examine practical, cost-effective weekday strategies to mitigate weekend discharge delays, focusing on Thursday and Friday planning as leverage points. According to recent studies, up to 15-20% of hospital stays are unnecessarily prolonged over weekends due to discharge barriers [1].

Instead of focusing solely on the weekend, we propose a shift in mindset, treating Thursday and Friday as critical decision-making days for discharge planning. These two days should be used to finalize consults, imaging, therapy needs, and post-discharge arrangements in anticipation of the weekend. Multidisciplinary teams can work together to proactively clear barriers, ensuring that patients are medically and logistically ready to leave the hospital even on a Saturday or Sunday.

In addition, avoidable weekend admissions such as social admits or low-acuity conditions can be reduced by embedding case management in the emergency department to help redirect patients toward timely outpatient follow-up, with appropriate contact numbers and support for scheduling appointments [3].

This review explores how structured pre-weekend planning can serve as a high-yield, resource-conscious strategy to reduce weekend-related delays and overall hospital length of stay.

## Review

Methodology

This narrative review examines the impact of weekend-related hospital delays on patient length of stay and evaluates practical interventions aimed at mitigating these effects. A literature search was conducted using PubMed, Google Scholar, and the Cochrane Library, focusing on English-language articles published between 2010 and 2024. Keywords included "weekend effect", "length of stay", "discharge planning", "hospital throughput", "seven day hospital coverage", and "multidisciplinary rounds".

The search initially retrieved 62 articles. Titles and abstracts were screened for relevance to the topic of hospital discharge timing and weekend-related delays. Full texts of potentially relevant studies were then reviewed, and 15 studies met the final inclusion criteria. Studies were included if they (1) involved adult inpatient hospital settings, (2) evaluated interventions addressing hospital length of stay, weekend discharge volume, or throughput efficiency, and (3) reported measurable outcomes relevant to discharge timing or patient flow. Priority was given to peer-reviewed studies, systematic reviews, multicenter quality improvement projects, and high-impact institutional reports, with most studies conducted in the United States or similar healthcare systems.

Studies were excluded if they (1) focused solely on pediatric or non-acute care populations, (2) were editorials, opinion pieces, or commentary articles without original data, (3) lacked measurable outcomes relevant to hospital discharge processes, or (4) examined unrelated outcomes such as solely financial analyses or patient satisfaction without discharge timing data.

Limitations of this narrative review include potential selection bias inherent to non-systematic reviews, lack of formal risk of bias assessment, and inability to synthesize pooled estimates due to heterogeneity in study designs.

This review synthesizes findings from published evidence to support a phased, weekday-focused strategy for reducing delays and optimizing discharge planning ahead of weekends.

The weekend effect and its operational impact

The weekend effect describes the observed reduction in hospital service availability during Saturdays and Sundays, resulting in care delays, prolonged hospitalizations, and lower discharge volume. Numerous studies have linked weekends to increased length of stay, partly due to limited access to consultants, imaging, procedures, and discharge planning support [1].

Hospitals functioning on a five-day operational model often see a buildup of medically ready patients who remain admitted over the weekend, occupying beds and straining throughput. These delays not only extend length of stay but also reduce capacity for new admissions, affect emergency department flow, and increase hospitalization costs [2]. The weekend effect is especially pronounced in diagnoses that require timely diagnostics or coordination with post-acute services, such as heart failure, stroke, or elective surgeries.

Limitations of expanding seven-day services

Expanding hospital services to provide full seven-day coverage is often proposed as a solution to weekend-related delays. While this model can improve patient flow in select units, its broad implementation across all departments is frequently cost-prohibitive. Studies have shown that outcomes from expanded weekend staffing, such as increased consultant availability, full diagnostic imaging access, and therapy services, are inconsistent in general medical and surgical wards, with limited gains in length of stay reduction [4]. Cost-effectiveness is more favorable in subacute rehabilitation settings but challenging to justify in routine inpatient care without substantial returns [5].

As a practical alternative, some hospitals have implemented partial weekend coverage, such as limited Saturday or Sunday shifts for allied health services. For instance, targeted weekend physiotherapy improved physical function in geriatric inpatients, facilitating discharge readiness in acute care settings [6]. Similarly, seven-day physical therapy coverage, including weekends, shortened the mean length of stay in a community hospital, with significant reductions for stroke and orthopedic patients [7]. These targeted approaches suggest partial weekend staffing can enhance discharge timing, though further research is needed to confirm their scalability across diverse hospital settings.

Thursday and Friday as strategic leverage points

To reduce weekend-related delays without expanding costly staffing, hospitals must reframe Thursday and Friday as high-leverage planning days. These two days offer the final full window to complete necessary diagnostic workups, finalize specialist recommendations, and arrange post-discharge services before weekend limitations take effect.

Prioritizing consults, imaging, and therapy orders on Thursday allows care teams to proactively remove barriers to discharge before Friday. On Friday, focused coordination efforts such as discharge huddles, case management reviews, and confirmation of follow-up can secure Saturday and Sunday discharges. A quality improvement project at a large US academic center demonstrated that front-loading discharge planning on Thursdays and Fridays increased weekend discharges by 12% and reduced the mean length of stay by 13% [8]. These findings suggest that a structured, weekday-centered approach can achieve similar results to seven-day staffing without the added costs. Additional reports have supported the idea that process redesign and weekday coordination improve discharge timing and mitigate the weekend effect [9].

Multidisciplinary rounds focused on pre-weekend barriers

Multidisciplinary rounds are a cornerstone of hospital discharge planning, bringing together physicians, nurses, case managers, therapists, and pharmacists to coordinate care and identify barriers to discharge. When these rounds are strategically focused on Thursday and Friday, they can play a pivotal role in preparing patients for timely weekend discharge.

By proactively addressing consult delays, pending imaging, or post-acute needs before the weekend, teams can avoid unnecessary prolongation of stay. Structured late-week multidisciplinary rounds have been associated with improved discharge readiness and better alignment across disciplines [10]. In large institutional quality projects, this approach has contributed to measurable reductions in length of stay and helped standardize discharge processes heading into the weekend [8]. Thursday and Friday should be approached with a distinct mindset, recognizing them not as ordinary weekdays but as two critical "anchor" days essential to ensuring patients are ready for discharge before the weekend. Prioritizing late-week collaboration creates momentum toward weekend discharges without relying on expensive staffing increases. Ultimately, this strategic approach not only optimizes resource utilization but also enhances patient experience by facilitating a smoother and more predictable transition of care [11].

Thursday rounds: identifying and preparing next-day discharges

On Thursdays, multidisciplinary teams should deliberately identify patients who are medically appropriate for discharge on Friday and proactively address any outstanding barriers. During Thursday rounds, the team can create a focused list of these patients, noting which laboratory tests, imaging studies, consultations, or discharge arrangements remain incomplete. This approach has been shown to improve weekend discharge rates and reduce hospital length of stay by enabling earlier and more predictable discharges [8]. Creating and using a structured checklist to track these patients and their pending needs further ensures that nothing is missed and that discharges can proceed smoothly on Friday morning [12].

Friday discharge huddles and checklist approaches

In addition to Thursday planning rounds, Friday should be treated as the final opportunity to confirm all elements of discharge readiness before the weekend. This includes not only patients planned for discharge on Friday but also those expected to leave on Saturday or Sunday. All necessary arrangements for weekend discharges, such as imaging, consultant recommendations, therapy assessments, medication reconciliation, discharge instructions, transportation, and follow-up appointments, should be fully completed on Friday. This ensures that weekend discharges can proceed smoothly, without needing additional input from case managers or ancillary staff during the weekend, and patients are not left waiting until Monday to go home.

The use of checklists during these huddles improves reliability and reduces the likelihood of missed tasks [13]. A 2010 study demonstrated that implementing a standardized discharge checklist increased on-time discharges and reduced overall length of stay [13]. Hospitals that adopt this type of pre-weekend coordination often report greater confidence in weekend planning and reduced pressure on limited weekend resources. Collectively, these findings demonstrate that structured checklists and Friday huddles strengthen discharge reliability and ensure readiness despite limited weekend resources.

Emergency department case management to reduce avoidable weekend admissions

Avoidable admissions that arise in the emergency department, often linked to low-acuity conditions, social concerns, or gaps in outpatient follow-up, represent a modifiable contributor to weekend census. Dedicated emergency department transitional care or case management teams have been shown to prevent unnecessary admissions in older adults and, more broadly, are the only intervention consistently associated with lower repeat emergency department use and downstream hospitalizations across multiple settings [14,15]. These teams can assist with real-time discharge planning, helping patients access outpatient testing, schedule follow-ups, and receive safety net resources without requiring hospital admission. Embedding case management in the emergency department, especially late in the week, may reduce Friday and Saturday boarding and decompress inpatient units ahead of the weekend. These findings reinforce the value of emergency department-based case management in managing inpatient census and preventing avoidable weekend admissions.

## Conclusions

Reducing weekend-related delays in hospitals does not always require a costly structural overhaul or full-scale expansion of services. While enhancing seven-day access to imaging, therapy, and consultants may be ideal, such approaches are often impractical for resource-constrained institutions. Instead, this review highlights a more immediate, high-yield alternative, treating Thursday and Friday as the most critical days of the hospital week.

By shifting the mindset toward proactive weekday coordination, hospitals can minimize weekend stagnation, free up beds faster, and create smoother discharge transitions. Thursday-focused multidisciplinary rounds allow teams to clear pending consults, imaging, and therapy orders before weekend limitations take hold. Friday discharge huddles and checklists improve reliability and ensure nothing is left to chance. Embedding case management in the emergency department adds a final layer, redirecting patients with low-acuity needs toward safe outpatient options and reducing avoidable weekend admissions.

This phased strategy is scalable, cost-conscious, and immediately actionable. It empowers frontline staff to create momentum heading into the weekend, breaks the cycle of Monday morning bottlenecks, and ultimately improves both patient flow and experience. Hospitals seeking to lower length of stay without expanding weekend staffing should consider making their Friday mindset their strongest discharge tool.
